# Effect of Bovine Follicular Fluid Small Extracellular Vesicles Isolated by Ultracentrifugation and Chromatography on In Vitro Oocyte Maturation and Embryo Development

**DOI:** 10.3390/ijms26072880

**Published:** 2025-03-22

**Authors:** Felipe Pérez-García, Erwin Muñoz-Acuña, Cecilia Valencia, Luis Aguila, Ricardo Felmer, María Elena Arias

**Affiliations:** 1 Laboratory of Reproduction, Centre of Reproductive Biotechnology (CEBIOR-BIOREN), Faculty of Medicine, Universidad de La Frontera, Temuco 4811230, Chile; f.perez12@ufromail.cl (F.P.-G.); erwin.munoz@ufrontera.cl (E.M.-A.); valencia.robles.cecilia@gmail.com (C.V.); luis.aguila@ufrontera.cl (L.A.); ricardo.felmer@ufrontera.cl (R.F.); 2 Doctoral Program in Sciences, Major in Applied Cellular and Molecular Biology, Faculty of Agriculture and Environmental Sciences, Universidad de La Frontera, Temuco 4811230, Chile; 3 Department of Animal Production, Faculty of Agriculture and EnvironmentalSciences, Universidad de La Frontera, Temuco 4811230, Chile; 4 Department of Agricultural Sciences and Natural Resources, Faculty of Agriculture and Environmental Sciences, Universidad de La Frontera, Temuco 4811230, Chile

**Keywords:** extracellular vesicles, follicular fluid, bovine, in vitro maturation, embryo development

## Abstract

Small extracellular vesicles (sEVs) play a crucial role in intercellular communication and have demonstrated significant relevance in reproductive biotechnology, particularly in in vitro maturation (IVM) and bovine embryo production. This study evaluates the effects of bovine follicular fluid-derived extracellular vesicles (ffsEVs) isolated using two methods: ultracentrifugation (UC) and size-exclusion chromatography (SEC) on oocyte maturation and preimplantational embryonic development. Significant differences in the size of ffsEVs obtained by both isolation methods were noted, with UC-derived ffsEVs (UC ffsEVs) being smaller than those isolated by SEC (SEC ffsEVs). UC ffsEVs were more effective in upregulating critical oocyte quality genes, such as *HSF1* and *CPT1B*. However, no significant differences were observed in embryonic developmental rates. Furthermore, the expression of genes associated with preimplantational embryonic quality revealed that only the SEC ffsEVs group exhibited a significant increase in *IFNT1* and *SOX2* levels, indicating an enhancement in embryonic quality. Notably, blastocysts derived from SEC ffsEVs also showed a higher total cell count compared to those from UC ffsEVs. No differences were found in other critical genes like *GLUT1* and *CDX2*. These results suggest that the use of SEC ffsEVs could improve the in vitro embryo production process, highlighting the importance of the isolation method in determining the functional efficacy of ffsEVs according to research objectives.

## 1. Introduction

In vitro embryo production (IVP) consists of three main stages: in vitro maturation of oocytes (IVM), in vitro fertilization (IVF), and in vitro embryo culture (IVC) [1]. Following decades of research and development, IVP has become the most successful assisted reproductive technique [2], enabling genetic improvement [3], species preservation [4], and the development of new reproductive technologies such as transgenesis and cloning [5]. However, in cattle, in vitro cultured embryos face several developmental challenges that render them inferior to their in vivo counterparts [6], including low blastocyst formation rates, altered cell numbers, higher apoptosis rates, abnormal gene expression, epigenetic alterations, and reduced resistance to cryopreservation [7,8,9]. The primary cause of these issues is the inability to develop an IVC environment that accurately replicates the in vivo conditions [10].

The gametogenesis, fertilization, and embryogenesis processes heavily rely on communication with cells, such as the bidirectional interaction within the cumulus–oocyte complex (COC) or between the trophectoderm and uterine epithelium [11,12] and the soluble elements present in the maternal environment, including hormones, growth factors, and cytokines, which are not fully replicated in the in vitro setting [10,13]. In this context, small extracellular vesicles (sEVs) have garnered significant interest over the past decade as mediators of intracellular communication. These nanoparticles, less than 200 nm in diameter and surrounded by a lipid bilayer, can originate as exosomes, which are formed within multivesicular bodies and released through exocytosis, or as microvesicles, which bud directly from the plasma membrane, and play a crucial role in cell-to-cell communication [14,15]. They serve as carriers for various biomolecules, including proteins, RNA, DNA, amino acids, and metabolites, facilitating the transfer of information and regulation between both proximal and distal cells [14,15]. Given the vesicles’ role in the organism, recent studies have proposed the application of sEVs as biomarkers and carriers for targeted delivery to modulate cellular metabolism and gene expression [16,17,18].

In animal reproduction, the involvement of sEVs in gamete and embryo development across various mammalian species has been highlighted. However, only a few studies have strategically explored the use of sEVs to enhance assisted reproductive techniques, such as in vitro embryo production. In cattle, small extracellular vesicles (sEVs) present in follicular fluid (ffsEVs), which are primarily secreted by granulosa, theca, and cumulus cells [19,20], are particularly noteworthy. These vesicles contain proteins and miRNAs associated with key pathways involved in oocyte maturation and embryonic development, including PI3K-Akt, EGFR, MAPK, ubiquitin-proteasome (UPS), and neurotrophin signaling pathways [21,22,23]. ffsEVs not only influence gene expression within cumulus–oocyte complexes and embryos [20,24,25,26] but have also been shown to stimulate primordial follicle activation, improve follicular development, enhance oocyte quality, and ultimately regulate processes favorable for oocyte competence and fertility outcomes [27,28]. Previous studies on the application of ffsEVs in in vitro embryo production have demonstrated significant changes in blastocyst formation [7,24,29], gene expression within cumulus–oocyte complexes and embryos, as well as stress resistance [30], varying according to the source of follicular fluid and the methods employed for vesicle isolation [31,32,33].

Thus, the aim of this study was to evaluate the impact of ffsEVs supplementation in the maturation medium, obtained through ultracentrifugation (UC) and size-exclusion chromatography (SEC), on oocyte and embryo quality through key indicators, such as blastocyst formation rate, total cell number, and transcript levels. A comprehensive exploration of UC- and SEC-derived ffsEVs aims to unravel nuanced differences in their effects on oocyte and embryo development. By analyzing these distinct isolation methods, we sought to elucidate potential variations in the bioactive cargo of ffsEVs and their consequential impact on reproductive outcomes. This approach could optimize maturation and in vitro production protocols and may have significant implications for reproductive health and fertility management in livestock.

## 2. Results

### 2.1. Isolation and Characterization of Small Extracellular Vesicles from Follicular Fluid (ffsEVs)

**Figure 1:**
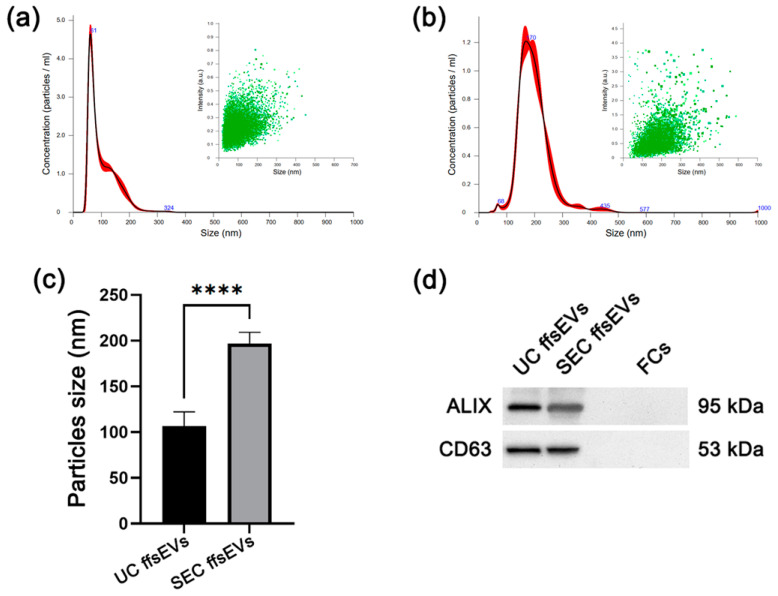
Characterization of small extracellular vesicles from follicular fluid (ffsEVs). (**a**) Size distribution, where black lines represent the mean particle distribution and the red areas indicate the standard deviation (SD), and intensity, where each green dot represents an event quantified by its size and intensity, of small extracellular vesicles isolated by ultracentrifugation (UC) and (**b**) size-exclusion chromatography (SEC). (**c**) Statistical comparison using Student’s *t*-test of particle sizes obtained through ultracentrifugation (UC ffsEVs) (106.801 ± 11.331 nm) and size-exclusion chromatography (SEC ffsEVs) (196.775 ± 9.368 nm). Significant differences are indicated by asterisks (**** = *p* < 0.0001). (**d**) Western blot of proteins obtained from UC ffsEVs, SEC ffsEVs, and follicular cells (FCs). The small extracellular vesicle markers ALIX and CD63 were positive in both ffsEVs samples and negative in FCs.

First, the ffsEVs isolated by ultracentrifugation (110,000× *g* for 70 min) (UC) and size-exclusion chromatography (SEC) were evaluated in terms of size using Nanoparticle Tracking Analysis (NTA). NTA analysis revealed an average vesicle size of 106.801 ± 11.331 nm for the UC isolation method (Figure 1a) and 196.775 ± 9.368 nm for the SEC method (Figure 1b), showing significantly different particle sizes between both isolation methods (Figure 1c).

**Figure 2:**
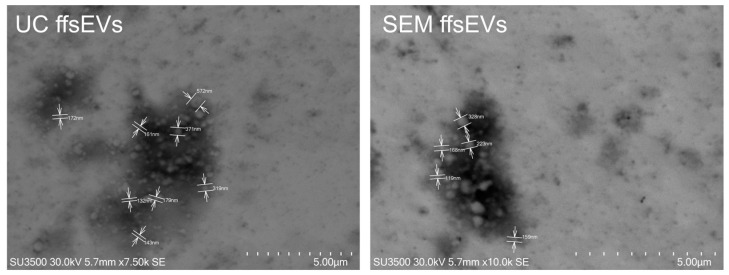
Scanning transmission electron microscopy (STEM) of small extracellular vesicles from follicular fluid isolated by ultracentrifugation (UC ffsEVs) and size-exclusion chromatography (SEC ffsEVs). A population of spherical sEVs with a size < 200 nm is observed in both samples. Medium and large extracellular vesicles (<200 nm) are also visible.

Western blot assays confirmed the presence of sEVs molecular markers CD63 and ALIX in both isolates, whereas no such markers were detected in the follicular cell (FCs) sample (Figure 1d). Electron microscopy analysis confirmed the predominant presence of small extracellular vesicles (sEVs) smaller than 200 nm. However, it was possible to observe extracellular vesicles of larger size (200–500 nm) as well (Figure 2).

**Figure 3:**
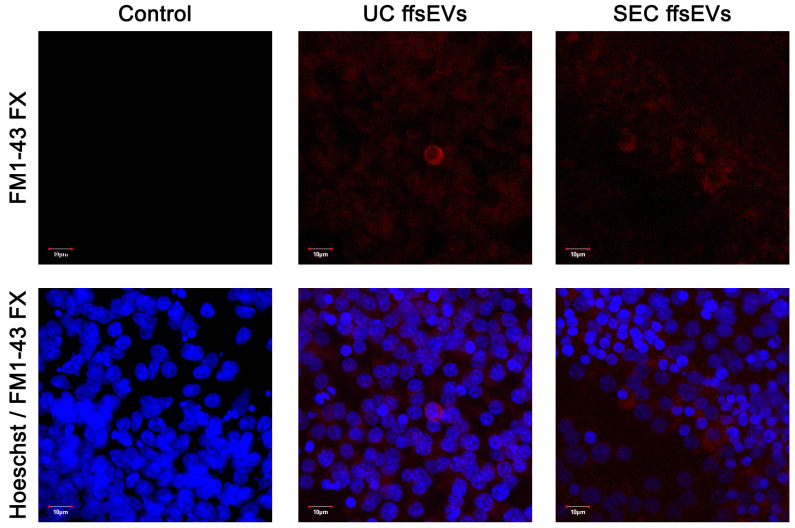
Confocal microscopy analysis of small extracellular vesicles from follicular fluid isolated by ultracentrifugation (UC ffEVs) and size-exclusion chromatography (SEC ffsEVs) and stained with FM1-43 FM (red). The nuclei of cumulus cells were labeled with Hoechst stain (blue). Scale bar: 10 µm.

For the ffsEVs uptake analysis, vesicles isolated by UC and SEC were stained with the lipid-specific marker FM1-43 FX and observed by confocal microscopy. The FM1-43 FX signal was only detected in the COCs exposed to ffsEVs during the in vitro maturation period (UC ffsEVs and SEC ffsEVs groups), with no signal observed in the Control group (Figure 3), indicating the ability of ffsEVs to internalize within cumulus cells.

### 2.2. Transcript Level Analysis of Mature Oocytes

**Figure 4:**
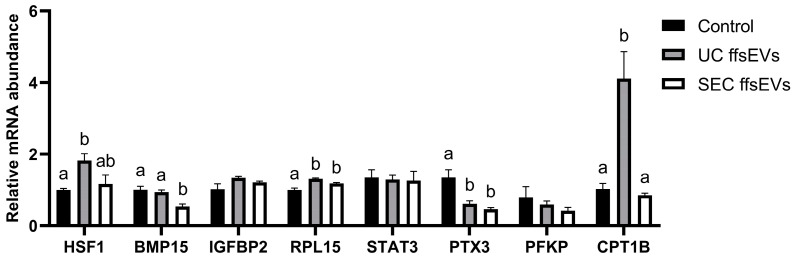
Effect of ffsEVs, isolated by ultracentrifugation (UC ffsEVs) and size-exclusion chromatography (SEC ffsEVs), supplementation in maturation media on the relative expression levels of bovine oocytes matured in vitro. The relative mRNA expressions of the genes *HSF1*, *BMP15*, *IGFBP2*, *RPL15*, *STAT3*, *PTX3*, *PFKP*, and *CPT1B* were obtained via RT-qPCR analysis, comparing the values 2^(−ΔΔCt)^ using analysis of variance (ANOVA). The results were normalized using the reference genes *HPRT1* and *GAPDH* expression levels. Statistically significant differences are indicated by groups of letters (a,b), where each letter represents a statistically homogeneous group, different letters indicate significant differences, and *p* < 0.05. Data are represented from 3 biological samples, each consisting of 30 mature oocytes.

Then, to evaluate the effect of ffsEVs during in vitro maturation on the oocyte transcript levels, relative expression analyses were performed on genes associated with the quality of mature oocytes. The results revealed a significant increase in the expression of the *HSF1* gene in the UC ffsEVs group compared to the Control group. An increase in the expression of *RPL15* was also observed in the UC ffsEVs and SEC ffsEVs groups compared to the Control group, along with an increase in *CPT1B* expression in the UC ffsEVs group. On the other hand, a significant decrease in *BMP15* expression was observed in the SEC ffsEVs group and *PTX3* expression in both the UC ffsEVs and SEC ffsEVs groups. No statistically significant differences were observed in the expression levels of *STAT3*, *PFKP*, or *IGFBP2* (Figure 4).

### 2.3. In Vitro-Fertilized Embryo Development

Oocytes matured in a medium supplemented with ffsEVs were fertilized in vitro to evaluate the effect of ffsEVs on the level of embryonic development. A total of 817 COCs, derived from four biological replicates per treatment, were fertilized in vitro and evaluated in terms of cleavage (day 3) and blastocyst formation (day 7). After evaluation, no significant differences were observed in embryonic development between the experimental groups. Likewise, there were no differences in the blastocyst development stage (Table 1).

### 2.4. Total Cell Number and Cell Localization

**Figure 5:**
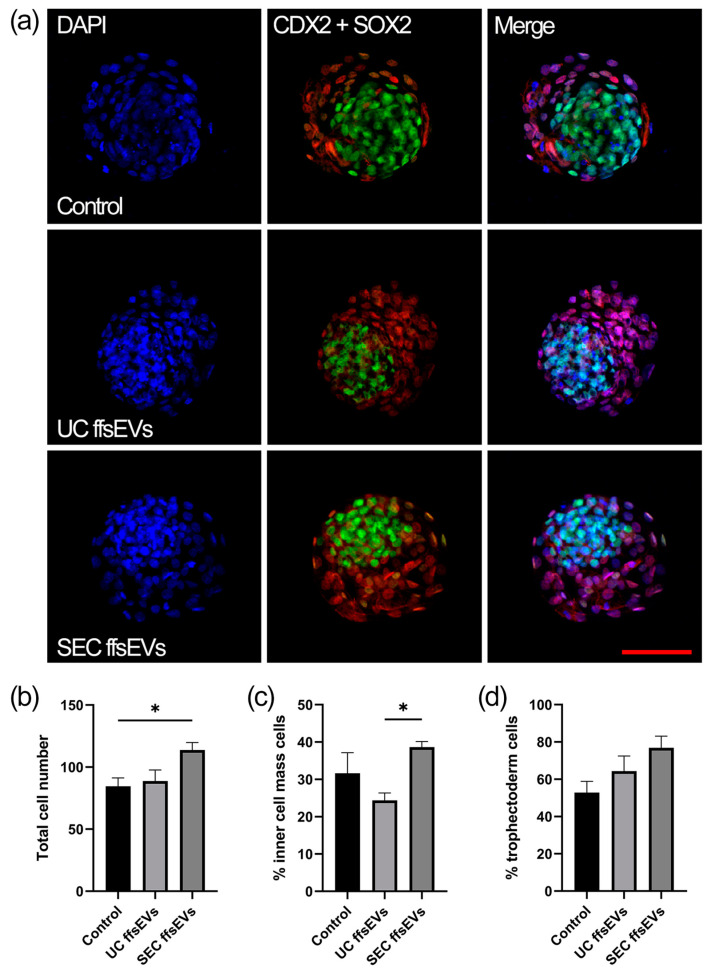
(**a**) Representative images of differential staining of blastocysts (n = 8) obtained from the Control oocytes and those treated with UC- and SEC-isolated ffsEVs and fertilized in vitro (IVF). The total cell number was evaluated using DAPI staining (blue); inner cell mass (ICM) cells were assessed by SOX2 immunostaining (green), and trophectoderm (TE) cells were evaluated by CDX2 immunostaining (red). The scale bar is the following: 100 µm. (**b**) The quantification of the total cells in blastocysts derived from the Control and UC- and SEC-FF-EV-treated oocytes and fertilized in vitro (IVF). (**c**) Percentage of ICM cells relative to the total number of cells. (**d**) Percentage of TE cells relative to the total number of cells. Statistical differences were determined by analysis of variance (ANOVA). *: *p* < 0.05.

Regarding the quality of the generated embryos, the total number of cells in the blastocysts was first quantified using DAPI staining (Figure 5a). The blastocysts obtained from the SEC ffsEVs treatment showed a significant increase in the total number of cells compared to those from the Control group and the UC ffsEVs treatment (Figure 5b). The inner cell mass (ICM) was evaluated using anti-SOX2 immunostaining (Figure 5a). A higher number of ICM cells was observed in the SEC ffsEVs treatment compared to the UC ffsEVs group (Figure 5c). Finally, the number of trophectoderm (TE) cells was analyzed using anti-CDX2 immunostaining (Figure 5a). No significant differences were found in the number of TE cells across the different treatments (Figure 5d).

### 2.5. Transcript Level Analysis of Embryos

**Figure 6:**
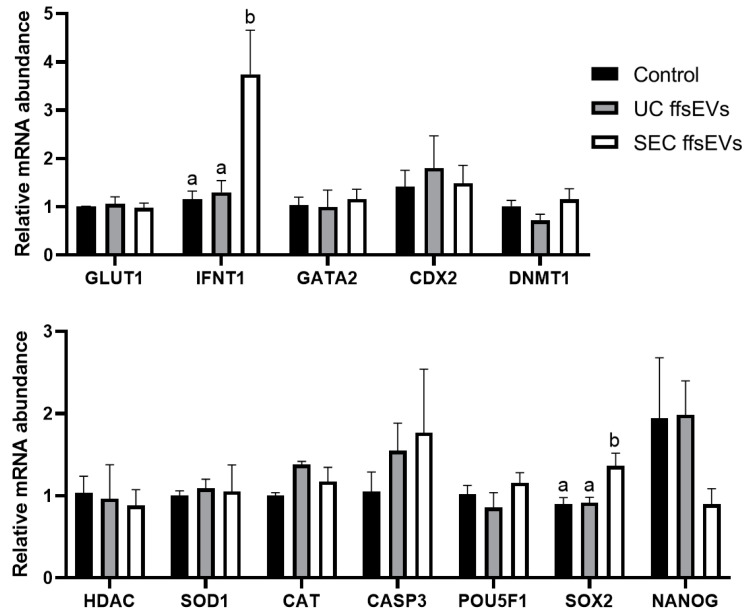
Effect of small extracellular vesicles isolated from follicular fluid by ultracentrifugation (UC) and size-exclusion chromatography (SEC) on the relative expression levels of bovine oocytes matured in vitro. The relative mRNA expressions of the genes *GLUT1*, *IFNT1*, *GATA2*, *CDX2*, *DNMT1*, *HDAC*, *SOD1*, *CAT*, *CASP3*, *POU5F1*, *SOX2*, and *NANOG* were obtained by RT-qPCR analysis, concerning to oocytes matured in vitro conventionally (Control), comparing 2^−(ΔΔCt)^ values through analysis of variance (ANOVA). The results were normalized based on the reference genes *SF3A1* and *HMBS* expression levels. The significant differences are indicated by letter groupings (a, b); each represents a statistically homogeneous group, while different letters indicate significant differences and *p* < 0.05. Data are represented from at least three biological samples, each consisting of groups of 5 blastocysts.

Finally, the effect of ffsEVs on the transcripts of genes associated with the quality of the embryos generated was evaluated. The results indicated changes only in the expression of the *IFNT1* and *SOX2* genes, which showed higher expression in SEC ffsEVs embryos compared to the UC ffsEVs treatment and the Control group (Figure 6). No statistically significant differences were observed in the expression levels of the genes *GLUT1*, *GATA2*, *CDX2*, *DNMT1*, *HDAC*, *SOD1*, *CAT*, *CASP3*, *POU5F1*, and *NANOG*.

## 3. Discussion

Small extracellular vesicles (sEVs) have emerged as a key factor in intercellular communication. These nanometric structures are enriched with various biomolecules, such as proteins, RNA, and lipids, which can influence cellular processes locally and distantly [14,23]. In the field of reproductive biotechnology, particularly in in vitro maturation (IVM) and in vitro production (IVP) of bovine embryos, extracellular vesicles derived from follicular fluid (ffsEVs) have attracted significant interest [20,23]. This study focused on evaluating the effect of ffsEVs obtained through the two most used isolation methods, ultracentrifugation (UC ffsEVs) and size-exclusion chromatography (SEC ffsEVs) [34], on oocyte maturation and embryonic development. Multiple factors, including transcript levels, embryo development rates, and blastocyst cell counts, were considered to better understand the effects of these ffsEVs.

Significant differences were observed in the size of ffsEVs isolated by ultracentrifugation (UC) and size-exclusion chromatography (SEC), corresponding to two heterogeneous groups, highlighting the importance of selecting the appropriate isolation method to maximize sEVs functionality. Additionally, UC ffsEVs exhibited a higher concentration, with the maximum value observed from a single sample being 2.53 × 10^11^ ± 1.83 × 10^9^ particles/mL, compared to SEC ffsEVs, which showed a maximum concentration of 1.93 × 10^10^ ± 4.71 × 10^8^ particles/mL. This result contrasts with previous reports from other authors where chromatography isolation yielded a higher particle count than ultracentrifugation [33,35,36]. The concentration of ffsEVs is particularly relevant for their use in supplementation during in vitro maturation (IVM) due to the availability of sufficient particles to effect cumulus–oocyte complexes (COCs) [29,37]. During the isolation process, the ffsEVs of both methods maintained their integrity and could be internalized by the cumulus cells, as demonstrated by the uptake assay using the FM1-43 FX marker.

Furthermore, cumulus cells play a critical role in transporting intracellular vesicles (IVs) to the oocyte through transzonal projections (TZPs). These IVs, which establish close contact with the zona pellucida and the oolemma, deliver mRNA and non-coding RNA crucial for the maternal RNA reserve, which supports early embryonic development [38]. While the direct uptake of EV cargo by oocytes has not been fully demonstrated in mammals, it is hypothesized that mRNA molecules in ffsEVs may be transferred indirectly to the oocyte through TZPs between cumulus cells and the oocyte [38,39]. For this reason, we decided to evaluate the transcriptional changes in mature oocytes following IVM supplementation with ffsEVs. Gene expression is a crucial indicator of oocyte maturation status and quality, and changes in the expression of specific genes can reflect the oocyte’s capacity for proper development and resilience to adverse conditions [40,41]. In this study, the levels of different transcripts associated with development and quality (*HSF1*, *BMP15*, *IGFBP2*, *RPL15*, *STAT3*, *PTX3*, *PFKP*, and *CPT1B*) [30] were evaluated in oocytes matured in vitro exposed or not to ffsEVs.

Initially, it was observed that *HSF1* levels, a transcription factor involved in regulating heat shock proteins (HSPs), significantly increased in oocytes treated with UC ffsEVs compared to other groups. *HSF1* assists in stress resistance by facilitating the synthesis, structural stability, degradation, and refolding of other intracellular proteins, depending on their condition [42]. In oocytes, *HSF1* is a maternal effect gene, the reduction or absence of which leads to early meiotic arrest in meiosis I or II and abnormal asymmetric divisions caused by defective spindle migration during cytokinesis [43,44]. Therefore, *HSF1* expression is essential during oocyte maturation and fertilization. Additionally, *HSF1* deficiency in embryos results in 1-cell stage arrest, mitochondrial damage, and altered redox homeostasis [45,46], making it critical for early embryo development. In this study, UC ffsEVs increased *HSF1* expression levels in matured oocytes compared to the Control group. This could suggest that UC ffsEVs may confer greater resilience to the stressful conditions oocytes face during fertilization and early embryo development.

Another gene relevant to oocyte maturation is *BMP15*, which has been related to the regulation of cumulus–oocyte complex energy metabolism and glucose uptake [47]. *BMP15* is expressed in oocytes and acts on follicular development, cumulus cell expansion, maturation, and ovulation [48,49]. In oocytes treated with SEC ffsEVs, a decrease in the levels of *BMP15* was observed compared to the other groups. Although *BMP15* supplementation has been shown to enhance in vitro maturation rates [50], the decrease in *BMP15* levels in the SEC ffsEVs group did not affect the maturation process, similar to what has been reported by [30], suggesting that while oocyte maturation remains unaffected, this effect could be related to reduced energy metabolism in COCs treated with SEC ffsEVs. Further studies focusing on this aspect are necessary to confirm these observations.

*RPL15* expression, a gene encoding ribosomal protein L15 involved in protein synthesis [51], was increased in both the UC- and SEC-isolated ffsEVs groups, suggesting a positive impact of ffsEVs on protein translation in oocytes. During oogenesis, the mRNA of ribosomal proteins, including *RPL15*, reaches peak levels in matured oocytes. Approximately 50% of these mRNAs are associated with polysomes throughout oogenesis, ensuring a consistent rate of ribosomal protein synthesis during later stages of oocyte development [52]. Decreased *RPL15* expression has been associated with accumulated defects in oocytes, compromising their developmental capacity and making them more likely to produce blastocysts with insufficient mass for expansion [53]. Future studies are needed to perform a functional analysis of translation levels in oocytes to further elucidate the role of *RPL15* and its contribution to oocyte quality.

On the other hand, the *PTX3* gene, synthesized by cumulus cells, has been widely described as a crucial factor in the synthesis of the extracellular matrix, which drives cumulus cell expansion during the maturation process [54,55,56]. However, its effect on oocyte competence and early embryo development remains unclear [57,58]. This study observed a decrease in *PTX3* levels in mature oocytes from both the UC- and SEC-isolated ffsEVs groups. Despite this decrease, no direct correlation between *PTX3* levels and cumulus expansion was observed in the oocytes treated with UC ffsEVs, suggesting that the regulation of this gene in oocytes may not be directly related to its function in cumulus cells or there might be compensation at the level of proteins secreted by cumulus cells. In this context, proteomic analyses would be essential to assess *PTX3* at the protein level and determine whether post-translational regulation could explain this finding.

Finally, a significant increase in the expression of the *CPT1B* gene was observed in oocytes treated with UC ffsEVs. *CPT1B* is a gene involved in lipid metabolism and plays a crucial role in regulating fatty acid oxidation, an essential process for oocyte maturation and resistance to vitrification-induced stress [59,60,61]. Previous reports indicate that in vitro-matured oocytes show lower levels of *CPT1B* transcripts than their in vivo counterparts [62]. Our results suggest that oocytes treated with UC ffsEVs may have a greater capacity to utilize lipids as an energy source during maturation, which could enhance their cryotolerance and their ability to withstand preservation techniques used in in vitro embryo production [61].

Regarding embryonic in vitro development, there were no significant differences between the experimental groups. Although the group treated with UC-ffsEVs presented a slight increase in the percentage of blastocysts, this increase did not reach statistical significance (Table 1). These results contrast with previous studies that have reported improvements in embryonic development following supplementation with ffsEVs in the maturation medium [24,29]. A possible explanation for this discrepancy could be related to the variability in the source of the oocytes used, as COCs obtained from local slaughterhouses may come from animals of varying ages, hormonal stimuli, and reproductive conditions, which could influence their intrinsic quality and their capacity to respond to ffsEV supplementation [25,26,63].

Regarding the expression analysis of genes associated with preimplantation embryonic quality (*GLUT1*, *IFNT1*, *GATA2*, *CDX2*, *DNMT1*, *HDAC1*, *SOD1*, *CAT*, *CASP3*, *POU5F1*, *SOX2*, and *NANOG*) [64], only the SEC ffsEVs group showed a significant increase in both *IFNT1* and *SOX2* compared to the Control group and UC ffsEVs. *IFNT1* plays a crucial role in embryonic development and maternal recognition of pregnancy, being most abundant in hatched blastocysts [65]. This correlates with the SEC ffsEVs group, which exhibited a tendency for a higher expanded proportion of blastocysts rate, though this difference was not statistically significant. The effect on *IFNT1* levels aligns with findings from other studies that supplemented embryo culture media with uterine fluid ffsEVs [66,67], suggesting that using SEC ffsEVs could enhance the implantation process of in vitro-produced embryos. While *GATA2* and *CDX2* are involved in regulating IFNT [68], in this study, no differences were observed in their expression among the groups, suggesting that the SEC ffsEVs may have acted directly with IFNT or influenced an upstream regulatory pathway independent of *GATA2* and *CDX2*.

*SOX2* is role critical in establishing and maintaining embryonic pluripotency [69,70]. *SOX2* expression begins at the 16-cell stage and becomes restricted to the inner cell mass (ICM) in bovine embryos [71]. The increase in *SOX2* expression in SEC ffsEVs blastocysts aligns with the higher observed ICM cell count of these embryos. Similarly to *IFNT1*, the increment in *SOX2* levels parallels findings where embryo culture media was supplemented with uterine fluid [66], suggesting that sEVs contribute with maternal factors crucial for embryonic development to the in vitro environment. While an increase in *SOX2* is generally favorable, as low expression levels lead to loss of pluripotency and a reduction in other related genes like *NANOG* [71,72], sustained overexpression of *SOX2* may result in embryonic arrest or cell death [73]. Additional research is needed to assess the behavior of this gene after day 7 of culture to determine its long-term impact on embryonic development.

Finally, evaluations of embryonic cell numbers showed a higher total cell count on day 7 of culture in the SEC ffsEVs blastocysts and a higher percentage of ICM cells in the SEC ffsEVs group. No significant differences between treatments were observed in the trophectoderm (TE) cell ratio. The total number of embryonic cells and their distribution (ICM and TE) are critical indicators of embryonic quality and developmental potential [74,75,76]. In bovines, higher quality is associated with blastocysts that exhibit a greater total cell count and ICM proportion within the normal range of 20 to 40% [77,78]. Blastocysts with an ICM ratio exceeding 40% are often linked to placental abnormalities or early fetal losses post-implantation [77]. Regarding cell count and gene expression, the embryos from the SEC ffsEVs group demonstrated better quality in terms of total cell number, suggesting that the presence of ffsEVs in an in vitro maturation environment may have significant implications for the initial stages of development. However, the precise underlying mechanisms responsible for the phenotypic and genetic changes of in vitro-produced bovine embryos require further elucidation.

## 4. Materials and Methods

### 4.1. Follicular Fluid Collection and Small Extracellular Vesicle (sEV) Isolation

Groups of 40 bovine ovaries were collected from pools of cows and heifers at a local slaughterhouse (Frigorífico Temuco, Temuco, Chile). Follicular fluid was obtained by aspirating 2 to 7 mm ovarian follicles using an 18 G hypodermic needle and syringe. The follicular content was allowed to settle for 10 min at 38.5 °C to precipitate cumulus–oocyte complexes (COCs) and tissue remnants.

ffsEVs were purified by ultracentrifugation and chromatography on size-exclusion columns. Ultracentrifugation was performed using the method previously described by da Silveira et al. (2017) [24], with slight modifications. Briefly, the follicular fluid was centrifuged at 300× *g* for 10 min to discard live cells, at 2000× *g* for 10 min to discard residual cells and cell fragments, and at 15,500× *g* for 30 min to discard apoptotic bodies and larger microvesicles. The supernatant was twice filtered through a sterile 0.22 μm filter and then stored at 4 °C for a maximum of 24 h. After that, the samples were transferred into 36 mL ultracentrifuge tubes completely filled with 1X DPBS saline solution (HyClone Laboratories, Inc., Logan, UT, USA). Subsequently, the samples were ultracentrifuged for 70 min at 110,000× *g* using a Beckman SW32Ti Rotor (Beckman Coulter, Inc, Brea. CA. USA) Afterward, the pellet was washed with DPBS under the same conditions in the ultracentrifuge. All the centrifugation steps were performed at 4 °C. Finally, the pellet of ffsEVs was resuspended in 200 μL of DPBS and stored at −80 °C for further use.

Isolation by size-exclusion chromatography was made using Izon qEv2 columns (IZON Science, Christchurch, New Zealand), following the manufacturer’s recommendations. Briefly, the columns were washed twice with 13.5 mL of 20% ethanol and twice with 13.5 mL of degassed DPBS. One milliliter of follicular fluid was loaded onto the column, and 700 µL fractions were collected and kept at −80 °C.

### 4.2. Nanoparticle Tracking Analysis (NTA)

The quantification of particle number and size distribution of isolated extracellular vesicles from follicular fluid was determined using Nanoparticle Tracking Analysis (NTA) with the NanoSight NS300 system (Malvern Technologies, Malvern, UK), following the method by Brennan et al. (2020) [79]. ffsEVs samples were diluted in particle-free DPBS at a ratio of 1:100 and analyzed at 22 °C, under constant flow (flow rate: 50), with a 488 nm laser and a camera level of 9. Data analysis was performed using NTA 3.4 software with a detection threshold and a size bin of 2.

### 4.3. Transmission Scanning Electron Microscopy (STEM)

For microscopy analysis, samples were diluted 1:250 in 4% (*v*/*v*) glutaraldehyde (Sigma-Aldrich, Burlington, MA, USA). Subsequently, a 5 μL droplet of the solution was applied to a carbon/formvar-coated copper grid with a 200-mesh size and incubated at room temperature for 10 min. Images were acquired using a SU3500 scanning electron microscope (Hitachi, Tokyo, Japan) with an acceleration voltage of 30 kV.

### 4.4. Total Protein Quantification and Western Blot

The lysis of EVs was performed using a 1:1 ratio of RIPA buffer for 30 min on ice. Since the RIPA buffer interferes with the Micro BCA quantification analysis (Soares Martins et al., 2018) [80], protein lysates were previously diluted 1:10 in DPBS. The BCA protein quantification assay was conducted on a microplate following the manufacturer’s instructions. Absorbance readings were quantified using an Infinite M200 NanoQuant plate reader (Tecan Group Ltd., Männedorf, Switzerland).

For the Western blot assay, a volume equivalent to 30 μg of protein from EV samples was lysed in 2× SDS buffer (Winkler, Santiago, Chile) containing 5% ß-mercaptoethanol (Sigma-Aldrich) for 5 min at 100 °C. The lysate was stored at −80 °C until the time of electrophoresis. Thirty microliters of protein lysate were loaded onto a 12% sodium dodecyl sulfate–polyacrylamide gel (SDS-PAGE) and ran at 110 V for 80 min. Wet transfer was performed to a polyvinylidene difluoride (PVDF) membrane (Millipore, Bedford, MA, USA) for 28 h at 50 mA. The membranes were immersed in methanol and air-dried for 30 min, followed by incubation with specific monoclonal antibodies for EVs surface marker CD63 (System Biosciences, Palo Alto, CA, USA. EXOAB-CD63A-1, 1:1000) and internal EVs marker ALIX (Thermofisher Scientific, MA1-83977, 1:1000) (Waltham, MA, USA) for 90 min [81]. Subsequently, membranes were incubated with HRP-conjugated anti-mouse secondary antibodies (Cell Signaling Technology, Danvers, MA, USA. 7076, 1:2000) for 30 min. Antibody binding was detected using the Westar Supernova chemiluminescence kit (Cyanagen, Bologna, Italy) following the manufacturer’s instructions and visualized through autoradiography (Amersham, Buckinghamshire, UK). All antibodies were diluted in a blot buffer composed of 1× TBS, 0.1% (*v*/*v*) Tween-20, and 1% (*m*/*v*) protease, fatty acid, and globulin-free BSA.

### 4.5. In Vitro Oocyte Maturation and ffsEVs Supplementation

Groups of 40 bovine ovaries were collected from a local slaughterhouse (Frigorífico Temuco, Temuco, Chile). Cumulus–oocyte complexes (COCs) were aspirated from follicles ranging from 2 to 7 mm. Then, COCs with multiple layers of compact cumulus cells were selected and cultured in TCM-199 medium supplemented with 10% inactivated FBS (Cytiva, Marlborough, MA, USA), 6 μg/mL LH hormone (Sioux Biochemical, Inc., Sioux City, IA, USA), 6 μg/mL FSH hormone (Bioniche Life Science Inc., Belleville, Ontario, Canada), and 1 μg/mL estradiol. The maturation medium for the treatment groups was supplemented with ffsEVs at a volume equivalent to 1 × 10^9^ particles/mL, while an equal volume of DPBS was used for the Control group. Finally, the COCs were incubated at 38.5 °C, 5% CO_2_, and saturated humidity for 24 h.

### 4.6. ffsEVs Uptake Assay

ffsEVs membranes were labeled with the FM^TM^ 1–43 FX dye (Thermofisher Scientific) following the manufacturer’s instructions [82]. After labeling, vesicles were washed twice in DPBS using a Vivaspin 20 centrifuge filter (Cytiva) at 2000× *g* for 5 min to remove excess staining from the medium. A volume of ffsEVs equivalent to 1 × 10^9^ particles/mL was added to the in vitro oocyte maturation medium under standard culture conditions [83]. The vehicle without ffsEVs served as the negative Control group.

For microscopic imaging, the procedures outlined by Arias et al. (2022) [84] with slight modifications were followed. Cumulus–oocyte complexes (COCs) were washed in PBS/PVP (0.1%) and fixed in a 4% paraformaldehyde solution for 15 min at room temperature. The samples were stained with 10 μg/mL Hoechst 33342 (Thermofisher Scientific). Finally, the COCs were mounted on glass slides with a drop of Dako mounting medium (Agilent Technologies, Santa Clara, CA, USA) and analyzed using a FluoView FV1000 confocal microscope (Olympus, Tokyo, Japan).

### 4.7. In Vitro Fertilization and Embryo Culture

In vitro fertilization (IVF) was performed according to Arias et al. (2015) [64]. In brief, COCs were fertilized with frozen commercial spermatozoa (Alta Genetics Inc., Balzac, AB, Canada) at 1 × 10^6^ sperm/mL, which was previously thawed at 38.5 °C and separated by Percoll gradient 45–90% (GE Healthcare Life Sciences, Chicago, IL, USA) in IVF-TL medium supplemented with 0.2 mM sodium pyruvate, 6 mg/mL fatty acid-free albumin (Sigma-Aldrich), 25 μg/mL gentamicin sulfate (Sigma-Aldrich), PHE (80 μM penicillamine, 40 μM hypotaurine, and 10 μM epinephrine), and heparin (Sigma-Aldrich). The IVF process was carried out at 38.5 °C, 5% (*v*/*v*) CO_2_, and saturated humidity for 20 h.

Subsequently, presumptive zygotes were denuded of cumulus cells by vortexing and placed in KSOM culture medium (Sigma-Aldrich) supplemented with 1% (*v*/*v*) BME medium with essential amino acids (Gibco, Douglas County, IL, USA), 1% (*v*/*v*) MEM medium with non-essential amino acids (Gibco), and 0.5 µg/mL FGF-2 (Sigma-Aldrich). Culture was performed at 38.5 °C with a gas mixture of 5% CO_2_, 5% O_2_, 90% N_2_, and saturated humidity. The cleavage rate was recorded on day 3 of culture and the blastocyst rate on day 7 under a stereomicroscope.

### 4.8. Quantitative Real-Time Reverse Transcription PCR (Two-Step RT-qPCR)

Genetic expression analyses were conducted on groups of 30 MII oocytes per biological replicate (3 biological replicates in total) and groups of 5 blastocysts per biological replicate (3 biological replicates in total). Total RNA was extracted using the PicoPure RNA Kit (Applied Biosystems, Waltham, MA, USA) according to the manufacturer’s instructions, followed by reverse transcription using the RevertAid First Strand cDNA Synthesis Kit (Thermo-Scientific) in a final volume of 20 µL. The reaction was incubated for 60 min at 45 °C, followed by 5 min at 70 °C. Subsequently, a qPCR reaction was performed using 4 µL of cDNA, 10 µL of Brilliant II SYBR Green (Agilent Technologies, Santa Clara, CA, USA), and 2 µL of RNase- and DNase-free water (Cytiva) in a final volume of 20 µL, supplemented with 5 µM specific primers for genes related to oocyte quality (*HSF1*, *BMP15*, *IGFBP2*, *RPL15*, *STAT3*, *PTX*, *PFKP*, and *CPT1B*, with *HPRT1* and *GAPDH* as Reference genes) [30] (Table 2) and embryo genes (*GLUT1*, *IFNT1*, *GATA2*, *CDX2*, *DNMT1*, *HDAC1*, *SOD1*, *CAT*, *CASP3*, *POU5F1*, *SOX2*, and *NANOG*, with *HMBS* and *SF3A1* as reference genes) [64,85] (Table 3). Initial denaturation was performed at 95 °C for 10 min, followed by 40 cycles of 95 °C for 20 s, annealing at 57 °C for 20 s, and elongation at 72 °C for 20 s. Results were analyzed using MxPro—MX3000P v4.10 software (Agilent) and GraphPad Prism v. 9 (GraphPad Software, San Diego, CA, USA).

### 4.9. Total Cell Number, Cell Localization, and Confocal Microscopy

Immunodetection was performed according to Águila et al. (2024) [86] with slight modifications. Expanded blastocysts (8 embryos in total per group from 3 biological replicates) were collected on day 7 of incubation (3 biological replicates and 8 embryos in total per treatment) and washed with PBS/PVA. The zona pellucida was removed using 0.2% pronase (Roche Life Science, Mannheim, Germany) and fixed with 4% paraformaldehyde (Sigma-Aldrich) for 15 min. Permeabilization was performed in PBS containing 1% Triton X-100 (Promega Corporation, Madison, WI, USA) for 30 min, followed by blocking in DPBS with 0.1% Triton X-100, 0.1% Tween-20 (Winkler) 1% BSA, and 5% goat serum (Gibco) for 2 h. After blocking, the embryos were incubated with primary antibodies anti-CDX2 (Abcam ab10305, 1:300) (Cambridge, UK) and anti-SOX2 (Abcam ab10005, 1:300) in blocking solution overnight at 4 °C. After washing 3 times for 10 min and 3 times for 20 min, the blastocysts were incubated with secondary antibodies anti-mouse (Life Technologies, Eugene, OR, USA, 1:200) and anti-rabbit (Cell Signaling Technology, Danvers, MA, USA 7076, 1:200) conjugated with Alexa Fluor 488 and 633, respectively, for 1 h at room temperature. The embryos were washed 3 times for 10 min and 3 times for 20 min and mounted on slides using Prolong Gold Antifade with DAPI (Life Technologies). Imaging was performed using confocal microscopy with a FluoView FV1000 microscope (Olympus, Tokyo, Japan), and ImageJ 1.54f software (National Institutes of Health, Bethesda, ML, USA) was used to count embryonic cells.

### 4.10. Statistical Analysis

Quantitative data are presented as means and standard deviation (±S.D.). Differences between the Control group, UC ffsEVs treatment, and SEC ffsEVs treatment were analyzed using one-way ANOVA. Post hoc analysis to identify specific group differences was performed using the Tukey test. Comparisons between the two groups, specifically for particle size differences between the UC and SEC methods, were analyzed using the Student’s *t*-test. Statistical significance was considered at *p* < 0.05. All experiments were performed with at least three biological replicates and analyzed using GraphPad Prism v. 9 (GraphPad Software, San Diego, CA, USA) and InfoStat Statistical Software v. 2020I (InfoStat, Córdoba, Argentina).

## 5. Conclusions

In conclusion, the use of extracellular vesicles derived from follicular fluid in the in vitro maturation of oocytes represents a promising area in reproductive biotechnology. This study highlights the distinct effects of follicular fluid small extracellular vesicles (ffsEVs) isolated through ultracentrifugation (UC) and size-exclusion chromatography (SEC) on bovine oocyte maturation and embryonic development. UC-derived ffsEVs demonstrated significant advantages in upregulating key genes such as *HSF1* and *CPT1B*, which are crucial for stress resistance and energy metabolism, thus having a more pronounced impact on oocytes. In contrast, SEC-derived ffsEVs showed a specific increase in genes like *IFNT1* and *SOX2*, which are vital for embryonic development and pluripotency, and a significant effect on blastocysts, including a higher total cell count. These findings emphasize the importance of the isolation method in determining the functional efficacy of ffsEVs, which may influence their potential applications in enhancing in vitro embryo production.

## Figures and Tables

**Table 1:** Embryonic development of oocytes matured in a medium supplemented with ffsEVs isolated by ultracentrifugation (UC ffsEVs) and size-exclusion chromatography (SEC ffsEVs). No. = number.

Treatment	COCs No.	Cleavage	Total Blastocysts	Expanded	Hatched
		No. and (%)	No. and (%)	No. and (%) ^1^	No. and (%) ^1^
Control	280	227 (81.07 ± 8.65)	71 (25.36 ± 5.05)	25 (35.21 ±12.06)	4 (5.63 ± 6.66)
UC ffsEVs	230	199 (86.52 ± 6.29)	81 (35.22 ± 7.35)	37 (45.68 ± 10.72)	2 (2.47 ± 3.76)
SEC ffsEVs	307	261 (85.52 ± 6.41)	89 (28.99 ± 5.68)	37 (41.57 ± 17.00)	9 (10.11 ± 10.84)

^1^ Percentages are based on the total number of blastocysts.

**Table 2:** List of primers used for qPCR of genes associated with mature oocyte quality. The directions of the forward and reverse sequences are indicated as F and R, respectively. Ta = Annealing temperature.

Gene	NCBI Code	Sequence (5′-3′)	Ta (°C)	Product Size (bp)
*HSF1*	NM_001076809.1	F: CCAGCAACAGAAAGTCGTCA	54	92
		R: GGGGGATCTTTCTCTTCACC		
*BMP15*	NM_001031752.1	F: ATCATGCCATCATCCAGAACC	53	73
		R: TAAGGGACACAGGAAGGCTGA		
*RPL15*	NM_001077866.2	F: CAAACGCCCAGTTCCTAAGG	55	76
		R: TCGAGCAAACTTGAGCTGGTT		
*CPT1B*	NM_001034349.2	F: TCTCCAGCAAGTTCTCCAGC	54	126
		R: ATCTCTCCAGCCCTTAGCCA		
*IGFBP2*	NM_174555.1	F: AGCATGGCCTGTACAACCTC	55	95
		R: GATCAGCTTCCCGGTGTTAG		
*STAT3*	NM_001012671.2	F: TGGCATAGCTTCCTCTGTAT	50	183
		R: CTTAGGGTCTCCTTCAACCT		
*PTX3*	NM_001076259.2	F: CATATGCCAGTTGGGAAGGT	51	139
		R: GCCTTCTCCAGTCTCCCTTT		
*PFKP*	NM_001193220.3	F: AAGCACGAGGAGTTCTGTGTC	55	122
		R: TCACACGTATCGGTGATGGT		
*HPRT1*	NM_001034035.2	F: TGCTGAGGATTTGGAGGAGG	54	154
		R: CAACAGGTCGGCAAAGAACT		
*GAPDH*	NM_001034034.2	F: GGAGCCAAACGGGTCATCATCTC	57	233
		R: GAGGGGCCATCCACAGTCTTCT		

**Table 3:** List of primers used for qPCR of genes associated with mature blastocyst quality. The directions of the forward and reverse sequences are indicated as F and R, respectively. Ta = Annealing temperature.

Gene	NCBI Code	Sequence (5′-3′)	Ta (°C)	Product Size (bp)
*GLUT1*	M60448.1	F: CCCAGGTGTTCGGCCTGGAC	59	214
		R: TGCAGGTCGCGGGTCACGTC		
*CDX2*	NM_001206299.1	F: GCCACCATGTACGTGAGCTAC	56	140
		R: ACATGGTATCCGCCGTAGTC		
*GATA2*	NM_001192114.3	F: GAGGACTGTAAGCGTAAAGG	54	140
		R: AAGAACCAAGTCTCCCCAT		
*DNMT1*	NM_182651.2	F: CGCATGGGCTACCAGTGCACCTT	58	312
		R: GGGCTCCCCGTTGTATGAAATCT		
*INFT1*	NM_001015511.4	F: TCATTCGGGCCAGGAGCCTG	57	108
		R: TGGCCCTGGTGCTGGTCAGC		
*CAT*	NM_001035386.2	F: ACCCTCGTGGCTTGCCAG	54	192
		R: ACTCAGGACGCAGGCCTCC		
*HDAC1*	NM_001037444.2	F: GGCTCTGACTCCTTGTCTGG	53	103
		R: GCATAGGCAGGTTGAAGCTC		
*POU5F1*	NM_174580.3	F: CGAAAGAGAAAGCGGACGGAG	53	178
		R: TTGATCGTTTGCCCTTCTGG		
*SOX2*	NM_001105463.2	F: TTTGTCCGAGACGGAGAAGC	55	146
		R: CTCCCGGCAGTGTGTACTTA		
*CASP3*	NM_001077840.1	F: TACTTGGGAAGGTGTGAGAAAACTAA	55	71
		R: AACCCGTCTCCCTTTATATTGCT		
*SOD1*	NM_174615.2	F: GCTGTACCAGTGCAGGTC	54	195
		R: CATGGACCACCATCGTGC		
*SF3A1*	NM_001081510.1	F: GCGGGAGGAAGAAGTAGGAG	57	125
		R: TCAGCAAGAGGGACACAAA		
*HMBS*	NM_001046207.1	F: CTTTGGAGAGGAATGAAGTGG	53	80
		R: AATGGTGAAGCCAGGAGGAA		

## Data Availability

The datasets and materials used and/or analyzed during the current study are available from the corresponding author upon reasonable request.
